# Diagnostic value of urinary mevalonic acid excretion in mevalonate kinase deficiency (MKD)

**DOI:** 10.1186/1546-0096-12-S1-P75

**Published:** 2014-09-17

**Authors:** Jerold Jeyaratnam, Nienke ter Haar, Monique de Sain-van der Velden, Mariëlle van Gijn, Joost Frenkel

**Affiliations:** 1 Department of Pediatrics, University Medical Center Utrecht, Utrecht, Netherlands; 2 Laboratory for Translational Immunology, University Medical Center Utrecht, Utrecht, Netherlands; 3 Department of Medical Genetics, University Medical Center Utrecht, Utrecht, Netherlands

## Introduction

Mevalonate kinase deficiency (MKD) is a rare hereditary autoinflammatory syndrome, characterized by recurrent fever episodes with gastrointestinal complaints, rash and arthralgia. The deficient mevalonate kinase activity leads to elevated mevalonic acid, which is excreted in the urine. Therefore, an elevated mevalonic acid excretion is suggestive of MKD. However, the diagnostic value of this analysis has not been investigated yet and remains unclear.

## Objectives

To investigate the diagnostic value of urinary mevalonic acid excretion in patients with suspected MKD.

## Methods

In this single center study, we retrospectively analyzed the results of all patients in whom both measurement of urinary mevalonic acid excretion and genetic analysis of the *MVK*-gene had been performed in the preceding 17 years. Mevalonic acid excretion was analyzed by using gas chromatography - mass spectrometry (GC-MS) and was expressed as mmol/mol creatinine and compared with age dependent reference values. The presence of two *MVK* mutations was considered as gold standard for the diagnosis of MKD.

## Results

The study included 63 patients (33 male, 30 female, aged: 0-36 year) with clinical features suggestive of MKD. Twenty-one patients had more than one assessment of mevalonic acid excretion.

Thirteen patients harboured two *MVK* mutations. These 13 MKD patients suffered predominantly from recurrent fever episodes (n=13), diarrhoea (n=13), abdominal pain (n=13), arthralgia (n=12), myalgia (n=10), stomatitis (n=10) and rash (n=9). Further, arthritis (n=6), seizures (n=3), mild mental retardation (n=2), dysarthria (n=1) and retinitis pigmentosa (n=1) were reported. The disease started within the first year of life in all MKD patients. Two out of 13 had at least one negative mevalonic acid excretion. In one patient, this measurement was performed during a febrile episode. Another patient had one normal mevalonic acid excretion alongside five elevated assessments.

Six patients had an elevated mevalonic acid excretion, but harboured no *MVK* mutations. At least two of these assessments were performed during a fever episode. Multiple urine analyses were performed; all of the six patients had discrepancies between the urine analyses. Urinary mevalonic excretion was elevated twice in two patients, while four patients had only one elevated assessment. Main symptoms were recurrent fever episodes (n=5), abdominal pain (n=2), neutropenia (n=1), hypotonia (n=1), dysmorphic features (n=1), mild mental retardation and mild ataxia (n=1). None of the remaining 44 patients with a normal mevalonic acid excretion had an *MVK* mutation.

This resulted in a sensitivity of 92%, a specificity of 88%, a positive predictive value of 67% and a negative predictive value of 98%.

## Conclusion

MKD seems very unlikely in patients with a normal mevalonic acid excretion, but it cannot be excluded completely. Therefore, detection of urinary mevalonic acid should not be mandatory before genetic testing. Nonetheless, a positive urinary mevalonic acid excretion requires *MVK* analysis to confirm the diagnosis MKD.

## Disclosure of interest

J. Jeyaratnam: None declared., N. ter Haar: None declared., M. de Sain-van der Velden: None declared., M. van Gijn: None declared., J. Frenkel Consultant for: NOVARTIS, Speaker Bureau of: SOBI.

